# Long COVID-Related Fatigue During Pregnancy: A Systematic Review

**DOI:** 10.7759/cureus.93877

**Published:** 2025-10-05

**Authors:** Aikaterini Dimitrakopoulou, Antigoni Sarantaki, Christina I Nanou, Vasiliki E Georgakopoulou, Chrysoula Taskou, Maria Chouli, Athina Diamanti

**Affiliations:** 1 Department of Midwifery, Faculty of Health and Care Sciences, University of West Attica, Athens, GRC; 2 Department of Pathophysiology, Laiko General Hospital, Athens, GRC

**Keywords:** fatigue, long covid-19, post-viral sequelae, pregnancy, sars-cov-2

## Abstract

Long sequelae of COVID-19 (Long COVID), or post-acute sequelae of SARS-CoV-2 infection, encompasses a wide range of persistent symptoms, with fatigue emerging as one of the most prevalent and disabling. Pregnant individuals may be uniquely susceptible to post-viral fatigue due to immunological and physiological adaptations during gestation. This review consolidates existing data regarding the prevalence, risk factors, and clinical implications of Long COVID-associated fatigue in pregnant individuals.

A narrative review was conducted of studies examining fatigue among pregnant individuals with confirmed SARS-CoV-2 infection. Key outcomes included fatigue prevalence, symptom persistence, associated risk or protective factors, and comparisons with non-pregnant populations. Across both the acute and post-acute stages of COVID-19, fatigue emerged as a consistently common symptom. Its prevalence and persistence varied significantly across studies, partly due to heterogeneity in assessment tools and follow-up durations. Severe acute illness, hospitalization, obesity, and smoking during pregnancy were linked to a higher risk of prolonged fatigue, whereas anosmia appeared to act as a potential protective factor. In contrast, comorbidities such as hypertension, diabetes, and lung disease were not significantly linked to fatigue risk. No consistent associations were found with maternal age or alcohol use. Long COVID-related fatigue presents a substantial burden in pregnancy, with implications for maternal health, quality of life, and postpartum recovery. Early recognition, individualized care strategies, and public health interventions targeting modifiable risk factors are essential to support this vulnerable population. Ongoing research is essential to uncover underlying mechanisms and guide evidence-based clinical management.

## Introduction and background

Since its emergence in December 2019, COVID-19 has presented substantial global challenges [1]. The virus’s rapid global spread led the World Health Organization (WHO) to declare a Public Health Emergency of International Concern on January 30, 2020, followed by its designation as a pandemic on March 11, 2020 [2]. By December 29, 2024, the WHO reported more than 777 million confirmed cases and over 7 million deaths worldwide, underscoring the ongoing public health impact of SARS-CoV-2 [3].

While the acute phase of COVID-19 initially captured most scientific and clinical attention, increasing concern has focused on the persistence of symptoms beyond the acute illness, a condition now widely referred to as Long COVID [4]. According to the WHO and the UK National Institute for Health and Care Excellence (NICE) definitions, Long COVID is characterized by new, ongoing, or relapsing symptoms that persist for ≥12 weeks after the initial SARS-CoV-2 infection and cannot be explained by alternative diagnoses [4]. The clinical presentation encompasses a broad spectrum of manifestations, most commonly including fatigue (often quantified using tools such as the Fatigue Severity Scale (FSS)), cognitive impairment (frequently assessed with the Montreal Cognitive Assessment (MoCA)), respiratory difficulties, and neuropsychiatric disturbances, such as anxiety and depression, all of which significantly impair quality of life [5-8]. Epidemiological evidence suggests that between 10% and 30% of individuals infected with SARS-CoV-2 develop Long COVID, with many experiencing symptoms for months or even years after the initial illness [5-7].

Among the wide spectrum of Long COVID manifestations, fatigue stands out as one of the most common, persistent, and disabling symptoms. Frequently reported by over half of affected patients, fatigue often exceeds the prevalence of respiratory, neurological, or cardiovascular sequelae [8-10]. It is not only a key driver of reduced quality of life and functional impairment, but also a symptom with significant socioeconomic implications due to prolonged absenteeism and decreased productivity [9].

The underlying mechanisms of post-viral fatigue remain incompletely understood, but hypotheses include chronic immune dysregulation, autonomic nervous system imbalance, mitochondrial dysfunction, endothelial activation, and persistent neuroinflammation [11,12]. In pregnant individuals, these processes may be further modulated by physiological and immunological adaptations unique to gestation, including shifts in immune tolerance, increased metabolic and oxygen demands, and endocrine alterations, potentially heightening susceptibility to prolonged post-viral sequelae [13,14].

Moreover, pregnancy-specific confounders may significantly contribute to the risk, severity, and persistence of fatigue in this context. Common conditions such as iron-deficiency anemia and vitamin B12 insufficiency impair oxygen transport and mitochondrial energy production, while sleep disturbances and mood disorders (depression and anxiety) disrupt neuroendocrine and inflammatory homeostasis. Additionally, postpartum fatigue, a multifactorial condition influenced by hormonal changes, lactation, and sleep disruption, may overlap with or exacerbate Long COVID-related fatigue [15,16]. Accounting for these overlapping mechanisms is essential to accurately interpret symptom prevalence and develop effective management strategies.

Despite the growing literature on Long COVID, evidence specific to pregnancy - particularly regarding fatigue - remains scattered, heterogeneous, and methodologically variable. Some studies suggest that pregnancy may increase susceptibility to post-viral fatigue [15,16], while others report no significant differences compared to non-pregnant individuals [17,18]. To address this gap, we conducted a systematic review of the available literature to synthesize evidence on the prevalence, risk factors, and clinical consequences of Long COVID-related fatigue among pregnant individuals.

In this systematic review, we first present an overview of the epidemiology and clinical presentation of Long COVID-related fatigue, then explore the biological and pregnancy-specific factors influencing its manifestation, and, finally, discuss clinical implications and research priorities to guide future management.

## Review

Materials and methods

Search Strategy

This systematic review was conducted following the 2020 Preferred Reporting Items for Systematic Reviews and Meta-Analyses (PRISMA) guidelines. This systematic review and meta-analysis were registered in the International Prospective Register of Systematic Reviews (PROSPERO), with ID number CRD42025639337.

Comprehensive searches were performed on PubMed/MEDLINE, Scopus, Google Scholar, Web of Science, and the WHO COVID-19 database, covering the period from January 2020 to September 2025. Titles and abstracts were independently screened by two authors (ADim and ADia). Manual searches were also conducted on Google Scholar, along with reference screening of relevant articles to ensure comprehensive coverage. To ensure thoroughness, grey literature was also reviewed. Sources included clinical trial registries, conference proceedings, and government reports, identifying studies that may not have been published in peer-reviewed journals.

The search strategy utilized a combination of key terms related to pregnancy, COVID-19, and post-viral conditions. Keywords included: “pregnant,” “gestation,” “gestational,” “postpartum,” “long-COVID,” “post-COVID,” “Long-COVID syndrome,” “post-COVID syndrome,” “post-acute sequelae of SARS-CoV-2 infection,” “PASC,” “persistent symptoms,” “long-term symptoms,” “long-term,” “COVID-19,” “SARS-CoV-2,” “fatigue,” and “post-exertional malaise.”

Inclusion and Exclusion Criteria

The inclusion criteria were as follows: (a) primary studies investigating Long COVID fatigue in women who experienced COVID-19 infection during pregnancy; (b) full-text articles available for review.

The exclusion criteria included: (a) systematic reviews, meta-analyses, editorials, conference papers, and case series/reports; and (b) primary studies focusing on populations that experienced COVID-19 outside of pregnancy or that did not report data on fatigue.

PRISMA Process

Two authors independently performed the literature search, screening, and data extraction, to ensure methodological rigor and reduce selection bias.

Identification: A total of 1,250 articles were identified through database searches (PubMed/MEDLINE, Scopus, Google Scholar, Web of Science, and the WHO COVID-19 database) using predefined search terms. An additional 50 articles were identified through manual searches and by screening references from relevant studies. After removing 300 duplicate records, 1,000 unique articles remained for screening.

Screening: Titles and abstracts of 1,000 articles were screened for relevance to the research question. A total of 750 articles were excluded based on the following reasons: (a) not related to Long COVID fatigue or pregnancy (n = 500), and (b) case reports, editorials, systematic reviews, or meta-analyses (n = 250). Therefore, 250 articles were assessed for full-text eligibility.

Eligibility: A full-text review was conducted for 250 articles. Of these, 240 articles were excluded for the following reasons: (a) population outside the scope (e.g., non-pregnant individuals) (n = 140); (b) lack of specific data on Long COVID fatigue (n = 70); and (c) insufficient data or methodological quality concerns (n = 30). A total of 10 studies were included in the systematic review.

**Figure 1:**
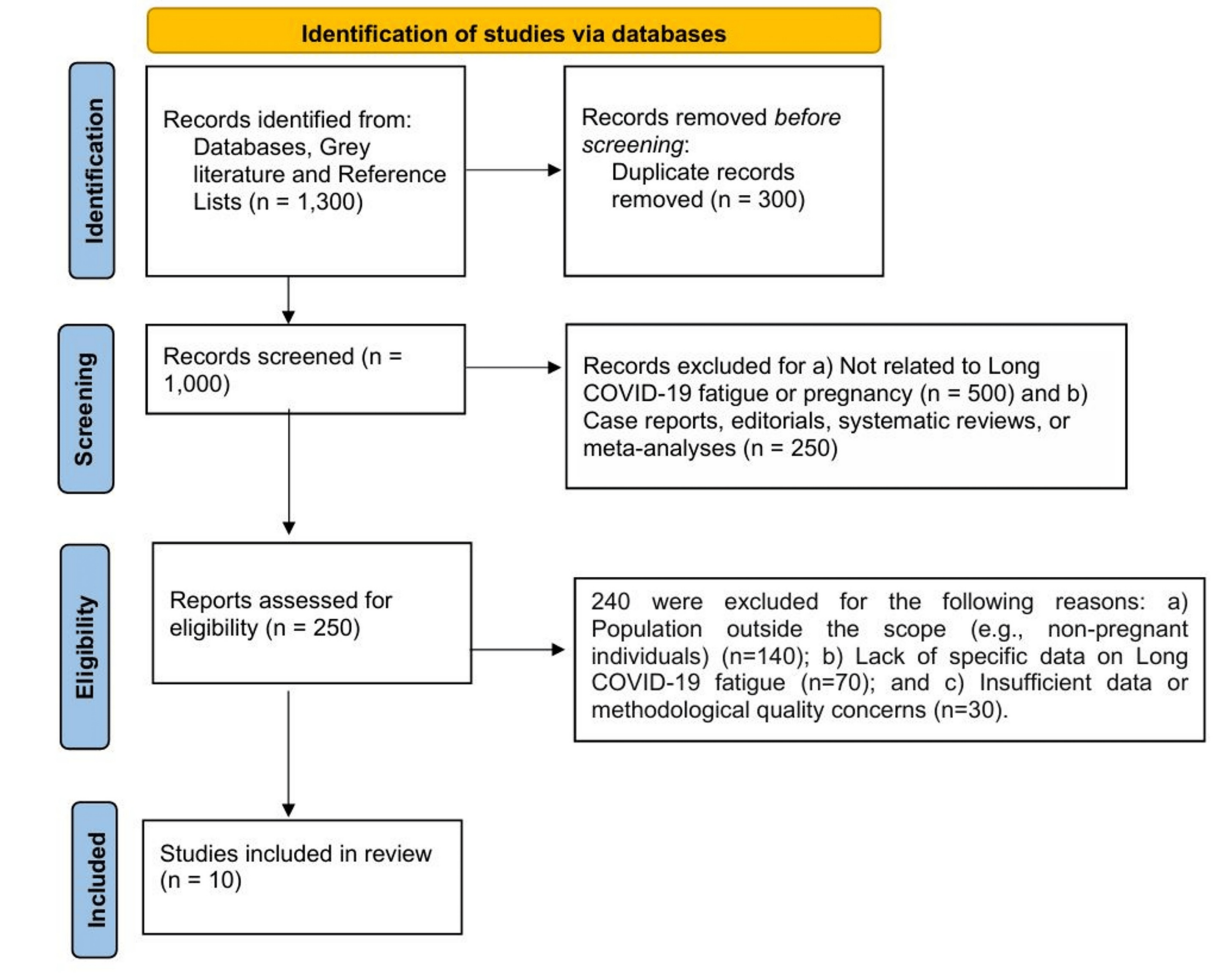
Flowchart of the study selection process.

The flowchart of the study selection process is illustrated in Figure 1.

Data Extraction

Data extraction was performed independently by two reviewers using a standardized form developed for this review. The data extraction template used in this systematic review is provided in Table 2 (see Appendix). For each included study, information was collected on the first author, publication year, country, study design, sample size, and key population characteristics, including maternal age, gestational stage at infection, and vaccination status when available. Details of fatigue assessment methods were recorded, including the use of validated instruments such as the FSS, Chalder Fatigue Scale, or EQ-5D-5L, as well as the timing of assessments during pregnancy, postpartum, or at follow-up intervals (e.g., six weeks, three months, six months, or longer). Data on the prevalence, severity, and duration of fatigue were extracted, along with reported risk factors such as severe acute COVID-19, hospitalization, obesity, and smoking, and protective factors such as anosmia. Co-occurring symptoms and key conclusions regarding the clinical significance of fatigue were also documented.

Quality Assessment

The quality of the studies included was independently evaluated by two reviewers using validated assessment tools. Cohort and case-control studies were assessed with the Newcastle-Ottawa Scale [19], which examines the domains of selection, comparability, and outcome. Each study received a score, with quality classified as high (7-9 stars), moderate (4-6 stars), or low (<4 stars).

Data Synthesis

A narrative synthesis was conducted to summarize variations in study design, population characteristics, measurement methods, and findings related to fatigue prevalence and associated risk factors. The heterogeneity among studies was explored qualitatively, focusing on differences in assessment tools, follow-up durations, and population demographics.

Results

Fatigue in the Context of Long COVID Syndrome

Fatigue is recognized as the most prevalent symptom during both the acute phase of COVID-19 infection [20] and the Long COVID syndrome phase [15,16,20,21]. 

In a longitudinal comparative study by Oliveira et al. [15], 25% of pregnant women diagnosed with COVID-19 developed fatigue six months post-infection. Fatigue was more common in pregnant patients with symptomatic COVID-19 compared to those who tested positive only at delivery or were not infected at all [15]. Similarly, a pilot study by Backes et al. [20] reported that 88% of women experienced fatigue during the acute phase of SARS-CoV-2 infection, ranging from mild (8.2%) to extreme (26.4%). Notably, 12 months postpartum, 46% of women continued to experience fatigue, with 30% describing it as very challenging or extremely burdensome. Experiencing severe symptoms in the acute phase of COVID-19 increased the risk of Long COVID among women, in contrast to those who remained asymptomatic.

In the study by Ghizzoni et al. [21], which followed 880 symptomatic individuals, 385 were COVID-19 positive, and 56 remained symptomatic during the study period. Of these, 30.4% exhibited persistent symptoms, including 29.4% lasting 30 days, 11.8% lasting three months, and 58.8% lasting six months or more. Fatigue was the most commonly reported symptom among those with symptoms persisting for at least six months (n = 6, 60%), followed by myalgia (n = 3, 30%), olfactory disorders (n = 3, 30%), and cough (n = 2, 20%). Most patients experienced multiple concurrent symptoms.

A prospective study identified fatigue as the second most common symptom in women who remained symptomatic four weeks after acute infection during pregnancy, affecting 15% of participants, with cough being the most common symptom (12%) [22]. Eight or more weeks post-infection, 25% of SARS-CoV-2-positive participants reported persistent symptoms. Fatigue (9.7%) remained the most prevalent symptom during this period, followed by cough (4.5%).

In a cross-sectional observational study by Belokrinitskaya et al. [23], the population was divided into two groups: pregnant women without comorbidities (n = 111) and non-pregnant women (n = 181). Fatigue affected 71.2% of pregnant women and 71.8% of non-pregnant women, with no significant differences in prevalence or severity between the groups. The authors attributed these findings to the participants’ young age and the absence of pre-existing somatic conditions.

According to Metz et al. [24], fatigue ranked as the second most common symptom of post-acute sequelae of SARS-CoV-2 (PASC), affecting 76.3% of patients, with post-exertional malaise being the most frequently reported symptom (77.7%).

A recent large-scale retrospective cohort study by Zang et al. further underscores the clinical significance of fatigue as part of the long-term sequelae of COVID-19 during pregnancy [25]. Using data from two national EHR networks (PCORnet and N3C), the authors found that among individuals infected during pregnancy, the incidence of post-acute sequelae involving cognitive, fatigue, and respiratory symptoms was 4.86 per 100 persons in PCORnet and 6.83 per 100 persons in N3C within 180 days after infection. Pregnant individuals had a significantly lower adjusted hazard of Long COVID compared with non-pregnant controls (aHR = 0.86, 95% CI: 0.83-0.90 in PCORnet), yet fatigue remained one of the most frequently coded components of the post-COVID syndrome. These findings add population-level evidence that fatigue continues to represent a core manifestation of Long COVID, even in the vaccination and Omicron era.

Association of Fatigue With Other Long COVID Symptoms

In the study by Oliveira et al. [15], which investigated post-viral fatigue following COVID-19 infection during gestation, it was observed that the presence of certain symptoms during the acute phase of infection increased the likelihood of experiencing persistent fatigue. Specifically, cough was associated with a significantly elevated risk (HR = 1.76; 95% CI: 1.07-2.96), as was myalgia (HR = 1.57; 95% CI: 1.01-2.44). Conversely, anosmia appeared to act as a protective factor, reducing the risk of prolonged fatigue (HR = 0.60; 95% CI: 0.40-0.88). These findings highlight the nuanced relationship between acute symptoms and long-term fatigue in the context of COVID-19 during pregnancy.

Association of Fatigue With Comorbidities

Among women infected with COVID-19 during pregnancy, no correlation was found between the duration of post-viral fatigue and other comorbidities or the trimester of pregnancy [15]. Additionally, the presence or number of maternal comorbidities, such as hypertension (HR = 1.15; 95% CI: 0.72-1.85), heart disease (HR = 0.89; 95% CI: 0.33-2.42), or lung disease (HR = 1.13; 95% CI: 0.65-1.95), did not independently influence the risk of fatigue [15]. Pathologies like diabetes and mental health disorders were also not observed among patients who experienced post-viral fatigue during pregnancy [15].

In contrast, according to Metz et al. [24], Long COVID was associated with risk factors such as obesity, pre-existing depression and anxiety disorders, economic hardship, and the need for oxygen therapy during the acute phase of infection. These findings underscore the complex interplay of individual and socioeconomic factors influencing the persistence of symptoms following COVID-19 infection.

Association Between Maternal Age and Long COVID Syndrome

According to studies by Oliveira et al. [15] and Kandemir et al. [16], there was no statistically significant difference in age between women who experienced Long COVID syndrome and those who did not. Similarly, in the cross-sectional observational study by Belokrinitskaya et al. [23], no notable variation in age was found between the groups of pregnant and non-pregnant women.

While fatigue appeared to persist longer in older women, this trend was not statistically significant in the study conducted by Oliveira et al. [15], suggesting that age alone may not be a decisive factor in the duration or severity of fatigue associated with Long COVID syndrome.

Association of Fatigue With Lifestyle Factors

According to Vásconez-González et al. [17], pregnant women who smoke are at a significantly higher risk of experiencing post-viral fatigue compared to non-pregnant women (OR = 114; 95% CI: 3.85-33.95). However, no statistically significant differences were observed between smokers and non-smokers regarding the overall symptoms of Long COVID syndrome.

Additionally, the frequency of fatigue occurrence was not influenced by alcohol consumption [17]. Among women who consumed alcohol, difficulty concentrating was reported as the most frequent symptom, though these findings were not statistically significant. In contrast, fatigue was the most common symptom among non-pregnant women who did not consume alcohol [17].

Table 1 summarizes all the studies providing evidence regarding Long COVID fatigue in pregnancy.

**Table 1:** Summary of findings regarding Long COVID fatigue in pregnancy. COVID-19: Coronavirus Disease 2019; CRONOS: COVID-19-Related Obstetric and Neonatal Outcome Study; EQ-5D-5L: EuroQoL 5 Dimensions, 5 Levels (a standard measure of health-related quality of life); G1: Group 1 (Symptomatic infection during pregnancy in this context); G2: Group 2 (COVID-19 positive at delivery in this context); G3: Group 3 (COVID-19 negative at delivery in this context); HER: Health Electronic Record; IPTW: Inverse Probability of Treatment Weighting (a statistical technique used to adjust for confounding); NIH: National Institutes of Health; PASC: Post-acute Sequelae of SARS-CoV-2; SARS-CoV-2: Severe Acute Respiratory Syndrome Coronavirus 2

Authors, Year	Country	Design and Study Population	Measurements	Key Findings for Fatigue	Period	Vaccination Rate	Fatigue Measurement	Follow-Up Times	Analysis/Covariates
Afshar et al. (2020) [22]	USA	Ongoing prospective cohort study (n = 594 pregnant or recently pregnant patients, COVID-19 positive or with suspected SARS-CoV-2 infection).	Self-reported questionnaires are filled in online, by phone calls, or by email when participants enroll and every week for 4 weeks. If applicable, questionnaires will then be administered at 24 and 32 weeks of pregnancy and 6 weeks, 6 months, and 12 months after pregnancy.	Fatigue was one of the most commonly reported symptoms among pregnant individuals with SARS-CoV-2 infection. It was present in 51% of cases during the acute phase and persisted in 25% of participants eight weeks or more after the onset of symptoms.	March-July 2020 (wild-type)	~0% (pre-vaccine)	Self-report checklist (non-validated)	Weekly ×4, then 24 w, 32 w (pregnancy) and 6 w, 6 m, 12 m postpartum (protocol)	Descriptive incidence; no multivariable modeling reported; no adjustment for covariates detailed
Oliveira et al. (2022) [15]	Brazil	Longitudinal comparative cohort study (n = 588) (3 groups: G1: 259 symptomatic infection during pregnancy, G2: 131 COVID-19 positive at delivery, G3: 198 COVID-19 negative at delivery). Pregnant women vaccinated for SARS-CoV-2 were not included.	Fatigue questionnaires at 6 weeks, 3 months, and 6 months. G1: 6 weeks, 3 months, 6 months after COVID-19 diagnosis, at delivery, and 6 weeks, 3 months, 6 months after delivery. G2 & G3: at delivery and 6 weeks, 3 months, 6 months after delivery.	The study found that post-viral fatigue was a common outcome among pregnant women with symptomatic SARS-CoV-2 infections during pregnancy, with prevalence rates of 40.6% at 6 weeks, 33.6% at three months, and 27.8% at 6 months after infection. The severity of the acute infection strongly influenced the risk and persistence of fatigue, with women who experienced severe COVID-19 being significantly more likely to report prolonged fatigue compared to those with mild disease. Key symptoms associated with an increased risk of persistent fatigue included cough and myalgia, while anosmia appeared to have a protective effect. Fatigue prevalence was significantly higher among women with symptomatic SARS-CoV-2 infection during pregnancy compared to those with asymptomatic infections or negative serology. At six months post-delivery, fatigue was reported by 16.4% of women who had symptomatic infections, compared to none in the asymptomatic group and only 2.9% in those with negative serology. Maternal age, comorbidities, and the trimester of pregnancy during infection did not significantly affect the risk or duration of fatigue.	~2020-2021 (ancestral/unknown variant)	0% (vaccinated excluded)	Custom fatigue questionnaire (binary symptom survey, not validated scale)	G1: 6 wk, 3 mo, 6 mo post-infection; plus at delivery & postpartum 6 wk, 3 mo, 6 mo. G2/G3: at delivery, and postpartum 6 wk, 3 mo, 6 mo	Cox regression for risk of persistent fatigue in G1; multivariate model including cough, myalgia; HR for moderate/severe vs mild disease
Vásconez-González et al. (2023) [17]	Ecuador	Cross-sectional study (n = 457) (33 pregnant, 424 non-pregnant)	Online 37-question questionnaire	The findings on fatigue from the study indicate that it was the most-reported Long COVID symptom, affecting 75% of pregnant women compared to 60.6% of non-pregnant women. However, this difference was not statistically significant. Pregnant women who smoked showed a higher risk of experiencing fatigue. Additionally, fatigue was more frequently reported among symptomatic participants during the acute phase of COVID-19.	April-July 2022 (Omicron variant)	Unknown (at least some vaccinated)	37-item self-report questionnaire (binary fatigue symptom)	Cross-sectional snapshot (no longitudinal follow-up)	Logistic/odds ratio models; smoking as a risk factor for fatigue; minimal covariate adjustment
Belokrinitskaya et al. (2023) [23]	Russia	Cross-sectional observational study (111 pregnant women, 181 non-pregnant women, both COVID-19 positive)	10-question questionnaire, comparison between the 2 groups	Fatigue occurred in 71.2% of the group of pregnant women and in 71.8% of the group of non-pregnant women. There were no differences found in both the frequency and the severity level between the pregnant and non-pregnant women. The authors mentioned that this result is likely attributable to their young age and the absence of any pre-existing somatic conditions.	July-October 2021 (Delta variant)	Not reported/unknown	Symptom questionnaire (0-10 severity rating) embedded in the 10-question PCS instrument	Single cross-sectional assessment (persistent symptoms, ≥4 weeks, ≥2 months)	Cross-sectional comparisons of frequencies/severities between groups; no multivariable adjustment reported
Kandemir et al. (2024) [16]	Turkey	Cross-sectional, retrospective study (n = 198) (99 COVID-19 positive pregnant women, 99 COVID-19 negative controls)	Data from medical records base and phone calls	The study found that fatigue was the most common symptom of Long COVID, reported by 54.5% of women with a history of SARS-CoV-2 infection during pregnancy. The likelihood of fatigue was significantly higher in symptomatic patients during the acute phase and those who required hospitalization. In comparison, only 8.1% of women in the control group, who did not have COVID-19, reported fatigue.	March 2020-April 2022 (wild type, Alpha, Delta, and Omicron variants)	Not reported/unknown	Symptom questionnaire (presence/absence) via survey	Single cross-sectional assessment (post-infection)	Logistic regression (multivariable) identifying predictors such as hospitalization, cough, myalgia; aORs reported, but the full covariate list was not detailed
Ghizzoni et al. (2024) [21]	Brazil	Retrospective cohort study (n= 880 pregnant women) (385 positive, 495 negative)	Data from medical records base and follow-up calls 3 and 6 months after	Fatigue was identified as the most frequent persistent symptom among pregnant women with COVID-19, reported by 60% of those with symptoms persisting for at least 6 months. It was part of a broader spectrum of post-COVID conditions observed in symptomatic patients, alongside other symptoms like myalgia and olfactory disorders. Most patients with persistent fatigue had experienced more severe symptoms during the acute phase of the illness.	March 2020-September 2023 (wild type, Alpha, Delta, and Omicron variants)	Not reported/unknown	Symptom persistence questionnaire/follow-up calls (presence of fatigue among persistent symptoms)	Follow-up at 3 and 6 months	Multivariate models for perinatal/maternal outcomes; symptom persistence described, but no clearly reported adjusted regression model for fatigue alone
Backes et al. (2024) [20]	Germany	Pilot study (n = 110 women) (of 773 women enrolled in the CRONOS registry)	Online questionnaire with questions on maternal symptoms and well-being during different periods postpartum (including fatigue and quality of life - EuroQoL EQ-5D-5L)	Fatigue was the most commonly reported symptom during the acute phase of COVID-19, experienced by 88% of participants. More than a year after the initial infection, fatigue persisted in 46% of participants, with 30% reporting it as very much or extremely challenging. Post-COVID symptoms, including fatigue, were more frequent in women who experienced severe COVID-19 during the acute phase. These findings highlight the significant long-term burden of fatigue in women infected with SARS-CoV-2 during pregnancy.	Infections April 2020-August 2021 (wild type, Alpha, and Omicron variants)	Not reported/unknown	Web-based symptom questionnaire (post-COVID survey matched with registry)	Single follow-up in December 2022-April 2023 (i.e., >1 year post-infection)	Descriptive frequencies, symptom comparisons by acute severity; no detailed multivariable model for fatigue reported
Bruno et al. (2024) [18]	USA	Retrospective cohort study (n= 89,312) (83,915 non-pregnant and 5,397 pregnant women, both groups were COVID-19 positive	HER data	The findings regarding fatigue from the study indicate that it was classified under the broader category of "malaise and fatigue" within the PASC infection. Among pregnant individuals with SARS-CoV-2 infection, the incidence of malaise and fatigue was 1.41%, compared to 4.68% in non-pregnant individuals.	March 2020- ~February/Mid 2022 (wild type, Alpha, Delta, and Omicron variants)	Not reported	“Malaise/fatigue” coded diagnosis within the PASC computable phenotype (EHR)	30 to 180 days post-infection	IPTW adjustment; adjusted hazard ratios comparing PASC incidence, adjusting for age, comorbidities, infection time, severity, etc.
Metz et al. (2024) [24]	USA	Multicenter prospective cohort study (n = 1,502 pregnant women)	NIH RECOVER - Pregnancy cohort, evaluation at least 6 months after infection using questionnaires.	76.3% of individuals diagnosed with PASC experienced fatigue as one of the most common symptoms. The study highlights fatigue as a prominent feature of PASC among participants at least 6 months after their initial infection during pregnancy, alongside other symptoms like post-exertional malaise and gastrointestinal disturbances​.	December 2021-September 2023 (61.1% infections ≥ December 2021, thus overlapping Omicron era)	51.4% fully vaccinated before infection	Symptom + severity composite scoring (PASC symptom score, threshold ≥12)	≥6 months post-infection (median ~10.3 months)	Multivariable logistic regression for PASC; covariates include obesity, mental health (depression/anxiety), economic hardship, acute oxygen therapy, vaccination status, among others
Zang et al. (2025) [25]	USA (PCORnet + N3C cohorts)	Retrospective cohort (pregnant women with SARS-CoV-2 in pregnancy, matched non-pregnant controls)	EHR-based infection and outcome data; computational phenotype definitions of Long COVID, including fatigue/malaise codes.	Among pregnant individuals infected with SARS-CoV-2, approximately 16.47% had Long COVID in PCORnet (using PCORnet CP) at 180 days. The incidence of the “cognitive, fatigue, and respiratory” cluster was approximately 4.86 per 100 persons in PCORnet. The adjusted hazard ratio (HR) comparing pregnant versus non-pregnant individuals was 0.86 (95% CI: 0.83-0.90; Nature)	March 2020-June 2023 (wild type, Alpha, Delta, and Omicron variants)	Not fully reported; less likely to be fully vaccinated in the pregnant group compared to the non-pregnant group, per baseline characteristics.	EHR diagnosis codes (“malaise/fatigue” within Long COVID phenotype)	Outcomes ascertained 30 days post-infection up to 180 days (i.e., 6 months)	Matching on region, age, infection time, acute severity, baseline comorbidities (diabetes, hypertension, autoimmune conditions, mental health, obesity, asthma).

**Figure 2:**
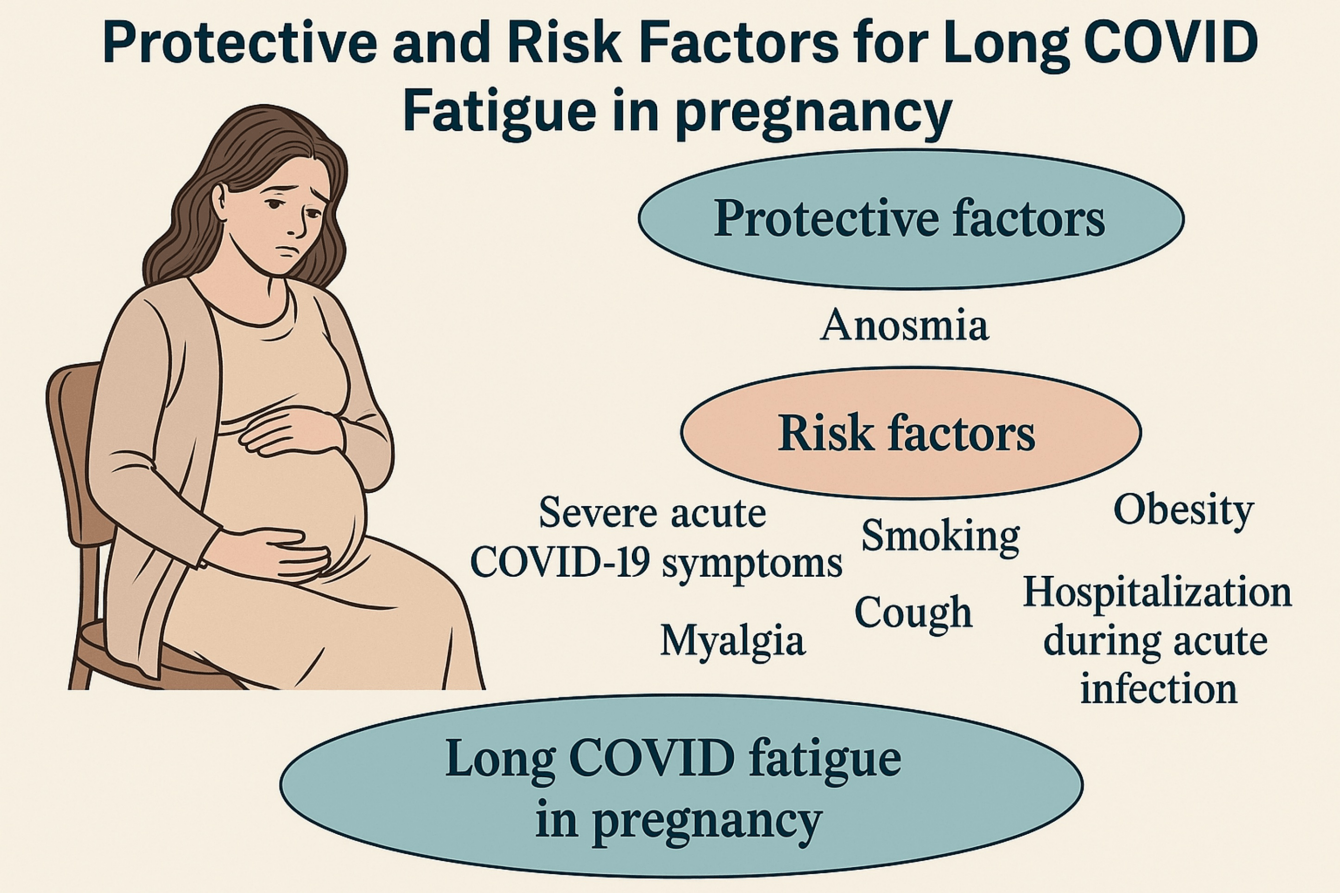
Risk and protective factors for Long COVID fatigue in pregnancy. The image was created by the authors of this article.

Figure 2 summarizes the risk and protective factors for Long COVID fatigue in pregnancy.

Clinical implications

Previous studies have identified fatigue as one of the most commonly reported symptoms during both the acute and post-acute phases of COVID-19 [15,16,20,21]. Pregnancy’s unique physiological and immunological adaptations may exacerbate the risk and severity of post-viral fatigue. Hormonal fluctuations, increased metabolic demands, and alterations in immune response during pregnancy could collectively predispose pregnant individuals to heightened vulnerability [26,27]. However, findings also indicate significant heterogeneity across studies, suggesting the influence of additional factors such as population characteristics, study designs, and measurement methods. For instance, some studies relied on subjective self-reported measures of fatigue, whereas others utilized standardized questionnaires, which may account for discrepancies in prevalence rates. Furthermore, variations in follow-up intervals (e.g., 6 months versus 12 months post-infection) likely contributed to differing fatigue persistence rates.

Pregnancy-specific factors, such as anemia, micronutrient deficiencies, mental health conditions, and sleep disturbances, may substantially modulate the risk, severity, and persistence of post-viral fatigue, yet these confounders have been insufficiently explored in the context of Long COVID. Iron and vitamin B12 deficiency, which are highly prevalent during pregnancy, can impair oxygen transport, mitochondrial function, and neuronal metabolism, thereby exacerbating fatigue and prolonging recovery following viral infections. These deficiencies not only contribute to baseline fatigue but may also interact synergistically with post-viral inflammatory processes, amplifying cytokine-mediated mitochondrial dysfunction and energy metabolism disturbances proposed as central mechanisms in Long COVID fatigue [9,11]. Moreover, anemia-induced hypoxia may enhance oxidative stress and impair skeletal muscle endurance, further compounding post-infectious asthenia. This physiological burden is compounded by increased iron demands during gestation and postpartum, suggesting that careful assessment and correction of nutritional deficiencies could be pivotal in mitigating fatigue severity and duration in this population.

Psychological and sleep-related factors, including antenatal and postpartum depression, anxiety, insomnia, and postpartum fatigue syndromes, represent additional, interlinked pathways that may heighten vulnerability to Long COVID-associated fatigue. Depression and anxiety are known to disrupt hypothalamic-pituitary-adrenal (HPA) axis regulation and autonomic balance, perpetuating chronic low-grade inflammation and neuroimmune activation - mechanistic hallmarks shared with post-viral fatigue syndromes [6,28]. Sleep disturbances, which affect up to 70% of pregnant individuals, further exacerbate inflammatory signaling and mitochondrial dysfunction, while impairing cognitive and physical recovery processes. Importantly, postpartum fatigue itself - driven by hormonal shifts, lactation demands, and disrupted sleep - may act as a confounding factor or even a mediator in the persistence of Long COVID fatigue. Collectively, these pregnancy-specific conditions underscore the multifactorial nature of post-viral fatigue and highlight the need for integrated clinical approaches that address nutritional, psychological, and sleep-related contributors alongside direct sequelae of SARS-CoV-2 infection.

Consistent with prior research, the presence of severe COVID-19 symptoms during the acute phase was identified as a significant predictor of prolonged fatigue [15]. Factors such as hospitalization, obesity, and smoking during pregnancy contributed to a heightened fatigue prevalence, underscoring the multifactorial nature of this condition. Interestingly, anosmia appeared to serve as a protective factor against prolonged fatigue, a finding that warrants further investigation into the underlying mechanisms [15].

In contrast, our review identified no significant association between fatigue and common comorbidities such as hypertension, diabetes, or lung disease [15]. These findings suggest that while some physiological stressors amplify fatigue risk, others may exert less influence than anticipated in the context of pregnancy and Long COVID.

The persistent nature of fatigue among pregnant individuals with Long COVID has significant clinical implications. Fatigue can severely impair quality of life, limiting functional capacity and contributing to psychological distress [28].

Healthcare providers should prioritize the early identification and management of fatigue in pregnant and postpartum individuals recovering from COVID-19. Comprehensive care strategies, including multidisciplinary support and tailored rehabilitation programs, may mitigate the impact of fatigue and improve outcomes. Additionally, public health interventions should address modifiable risk factors such as obesity and smoking, which were identified as contributors to fatigue severity [29]. These findings highlight the importance of promoting healthier lifestyles and providing targeted support to vulnerable populations during and after the COVID-19 pandemic.

## Conclusions

Fatigue is one of the most persistent and debilitating symptoms experienced by pregnant individuals with Long COVID, with multifactorial influences contributing to its onset and severity. While pregnancy-related physiological changes may heighten vulnerability to post-viral fatigue, our synthesis of the current literature highlights significant variability in prevalence estimates due to differences in study design, follow-up duration, and assessment tools. The severity of acute COVID-19 illness, along with modifiable factors such as obesity and smoking, was consistently associated with prolonged fatigue, whereas traditional comorbidities like hypertension or diabetes were not. Importantly, the absence of a clear association with age or many common chronic conditions points to the need for a more tailored approach to assessing risk. Given the potential impact on maternal well-being and perinatal outcomes, early recognition and multidisciplinary management of fatigue in this population should be prioritized. Future research should aim to clarify underlying mechanisms and guide tailored interventions to mitigate the long-term burden of fatigue in pregnancy-associated Long COVID.

## Appendices

**Table 2:** Extraction template of the systematic review.

Field	Description
Study ID	Author(s), year
Country	Country or region where the study was conducted
Study Design	Longitudinal cohort/cross-sectional/retrospective cohort/case-control
Sample Size	Total participants and subgroup breakdown (pregnant vs. non-pregnant, symptomatic vs. asymptomatic)
Population Characteristics	Age range, trimester of pregnancy, vaccination status if reported
Fatigue Assessment Method	Tool used (e.g., FSS, Chalder, EQ-5D-5L), clinical records, follow-up calls, or online questionnaires
Timing of Assessment	During pregnancy/postpartum/follow-up intervals (e.g., 6 weeks, 3 months, 6 months, ≥12 months)
Prevalence of Fatigue	Percentage and number of participants reporting fatigue at each time point
Severity of Fatigue	Mild/moderate/severe, if reported
Duration of Fatigue	Time frame over which fatigue persisted
Associated Risk Factors	e.g., severe acute COVID-19, hospitalization, obesity, smoking
Protective Factors	e.g., anosmia, mild acute infection
Other Long COVID Symptoms	e.g., cognitive impairment, dyspnea, myalgia
Key Conclusions	Authors’ main findings relevant to post-viral fatigue in pregnancy
Quality Assessment (NOS)	Number of stars (0-9) and quality classification (high, moderate, low)

## References

[1] Lai CC, Shih TP, Ko WC, Tang HJ, Hsueh PR (2020). Severe acute respiratory syndrome coronavirus 2 (SARS-CoV-2) and coronavirus disease-2019 (COVID-19): the epidemic and the challenges. Int J Antimicrob Agents.

[2] Damaskos C, Garmpi A, Georgakopoulou VE (2020). COVID-19: do it like Greece. Why Greece is coping with COVID-19 better than other countries?. Pan Afr Med J.

[3] World Health Organization (WHO) (2025 (2025). WHO COVID-19 dashboard. https://data.who.int/dashboards/covid19/deaths?n=o.

[4] Nalbandian A, Sehgal K, Gupta A (2021). Post-acute COVID-19 syndrome. Nat Med.

[5] Georgakopoulou VE, Makrodimitri S, Gkoufa A (2024). Lung function at three months after hospitalization due to COVID‑19 pneumonia: comparison of alpha, delta and omicron variant predominance periods. Exp Ther Med.

[6] Efstathiou V, Stefanou MI, Demetriou M (2022). Long COVID and neuropsychiatric manifestations (review). Exp Ther Med.

[7] Kampouridou K, Georgakopoulou VE, Chadia K, Nena E, Steiropoulos P (2024). Evaluating sleep disturbances in patients recovering from COVID-19. Pneumon.

[8] Karampitsakos T, Sotiropoulou V, Katsaras M (2022). Post-COVID-19 interstitial lung disease: insights from a machine learning radiographic model. Front Med (Lausanne).

[9] Abbott Z, Summers W, Niehaus W (2023). Fatigue in post-acute sequelae of coronavirus disease 2019. Phys Med Rehabil Clin N Am.

[10] Vu QM, Fitzpatrick AL, Cope JR (2024). Estimates of incidence and predictors of fatiguing illness after SARS-CoV-2 infection. Emerg Infect Dis.

[11] Liu Y, Gu X, Li H, Zhang H, Xu J (2023). Mechanisms of long COVID: an updated review. Chin Med J Pulm Crit Care Med.

[12] Lempesis IG, Georgakopoulou VE, Reiter RJ, Spandidos DA (2024). A mid‑pandemic night's dream: melatonin, from harbinger of anti‑inflammation to mitochondrial savior in acute and long COVID‑19 (review). Int J Mol Med.

[13] Abu-Raya B, Michalski C, Sadarangani M, Lavoie PM (2020). Maternal immunological adaptation during normal pregnancy. Front Immunol.

[14] Georgakopoulou VE, Taskou C, Spandidos DA, Sarantaki A (2025). Long COVID‑19 and pregnancy: a systematic review. Biomed Rep.

[15] Oliveira AM, Carvalho MA, Nacul L (2022). Post-viral fatigue following SARS-CoV-2 infection during pregnancy: a longitudinal comparative study. Int J Environ Res Public Health.

[16] Kandemir H, Bülbül GA, Kirtiş E, Güney S, Sanhal CY, Mendilcioğlu İİ (2024). Evaluation of long-COVID symptoms in women infected with SARS-CoV-2 during pregnancy. Int J Gynaecol Obstet.

[17] Vásconez-González J, Fernandez-Naranjo R, Izquierdo-Condoy JS (2023). Comparative analysis of long-term self-reported COVID-19 symptoms among pregnant women. J Infect Public Health.

[18] Bruno AM, Zang C, Xu Z (2024). Association between acquiring SARS-CoV-2 during pregnancy and post-acute sequelae of SARS-CoV-2 infection: RECOVER electronic health record cohort analysis. EClinicalMedicine.

[19] Wells GA, She B, O’Connell D, Peterson J, Welch V, Losos M, Tugwell P (2014). The Newcastle-Ottawa Scale (NOS) for Assessing the Quality of Nonrandomised Studies in Meta-Analyses. http://www.ohri.ca/programs/clinical_epidemiology/oxford.asp.

[20] Backes C, Pecks U, Keil CN (2024). Post-COVID in women after SARS-CoV-2 infection during pregnancy - a pilot study with follow-up data from the COVID-19-Related Obstetric and Neonatal Outcome Study (CRONOS). Z Geburtshilfe Neonatol.

[21] Ghizzoni AP, Santos AK, de Braga RS, Duz JV, Bouvier VD, de Souza MS, Silva DR (2025). Clinical characteristics, outcomes and persistent symptoms of pregnant women with COVID-19: a retrospective cohort study. Int J Gynaecol Obstet.

[22] Afshar Y, Gaw SL, Flaherman VJ (2020). Clinical presentation of coronavirus disease 2019 (COVID-19) in pregnant and recently pregnant people. Obstet Gynecol.

[23] Belokrinitskaya TE, Frolova NI, Mudrov VA, Kargina KA, Shametova EA, Agarkova MA, Zhamiyanova CT (2023). Postcovid syndrome in pregnant women. Obstet Gynecol.

[24] Metz TD, Reeder HT, Clifton RG (2024). Post-acute sequelae of severe acute respiratory syndrome coronavirus 2 (SARS-CoV-2) after infection during pregnancy. Obstet Gynecol.

[25] Zang C, Guth D, Bruno AM (2025). Long COVID after SARS-CoV-2 during pregnancy in the United States. Nat Commun.

[26] Ferrer-Oliveras R, Mendoza M, Capote S, Pratcorona L, Esteve-Valverde E, Cabero-Roura L, Alijotas-Reig J (2021). Immunological and physiopathological approach of COVID-19 in pregnancy. Arch Gynecol Obstet.

[27] Muñoz-Chápuli Gutiérrez M, Prat AS, Vila AD (2024). Post-COVID-19 condition in pregnant and postpartum women: a long-term follow-up, observational prospective study. EClinicalMedicine.

[28] Vélez-Santamaría R, Fernández-Solana J, Méndez-López F (2023). Functionality, physical activity, fatigue and quality of life in patients with acute COVID-19 and Long COVID infection. Sci Rep.

[29] Wang Y, Su B, Alcalde-Herraiz M (2024). Modifiable lifestyle factors and the risk of post-COVID-19 multisystem sequelae, hospitalization, and death. Nat Commun.

